# SURGICAL GASTROSTOMY BASED ON ENDOSCOPIC CONCEPTS

**DOI:** 10.1590/0102-6720201600010013

**Published:** 2016

**Authors:** Emmanuel Conrado SOUZA

**Affiliations:** Surgical Clinic, Santa Casa de Misericórdia de Itabuna and State University of Santa Cruz, Ilhéus, BA, Brazil

**Keywords:** Gastrostomy, Laparotomy, Enteral nutrition, Surgical technique

## Abstract

***Background*:**

Until the early 1980s, Stamm technique was considered standard method to gastrostomy. After description of the endoscopic technique, due to its efficiency and speed, quickly became the method of choice for long-term enteral access.

**Aim:**

Describe a technique that combines direct view of the stomach from open surgery with the simplicity and less traumatic endoscopic gastrostomy method.

***Method*:**

In patient supine under spinal anesthesia the technique stars with small epigastric incision to pull up the stomach. A 3 mm incision in the left hypochondrium is made to pass needle puncture to guidewire passage. The stomach is drilled, guidewire is seizured, connection to catheter and percutaneous approach is made with traction of the stomach to the abdominal wall. Purse suture on the anterior gastric wall is not needed.

***Results*:**

Twenty-eight patients underwent gastrostomy using endoscopy devices; six had local minor complications without the need for re-intervention; there was no death.

***Conclusion*:**

The surgical gastrostomy with minimal incision in the stomach to pull off the catheter using endoscopic gastrostomy devices, proved to be safe, easy to perform, less traumatic, quick, simple and elegant.

## Abstract (pt)

***Racional*:**

Até 1980, a técnica de Stamm era padrão para gastrostomia. Após introdução da técnica por via endoscópica, devido a sua eficiência e rapidez, rapidamente tornou-se método de escolha para acesso enteral prolongado.

**Objetivo:**

- Descrever uma técnica que combina a visualização direta do estômago pela laparotomia com a simplicidade e menor trauma oferecida pela gastrostomia endoscópica.

**Método:**

A técnica é laparotômica com o paciente em decúbito dorsal sob raquianestesia. Inicia-se com incisão pequena no epigástrio e tração do estômago através dela. Associa-se incisão de 3 mm em flanco esquerdo para punção com agulha permitindo a passagem de fio guia. A seguir, realiza-se perfuração punctiforme do estômago, apreensão do fio guia, conexão com sonda e tração percutânea com aproximação do estômago à parede abdominal sem necessidade de sutura em torno da sonda.

**Resultados:**

Vinte e oito pacientes foram submetidos à essa técnica com dispositivos usados pela endoscopia; seis apresentaram complicações locais menores, sem necessidade de reintervenção; não houve óbito.

**Conclusão:**

A gastrostomia cirúrgica com incisão mínima no estômago para exteriorização da sonda, utilizando dispositivos da gastrostomia endoscópica, mostrou-se segura, de fácil execução, menos traumática, mais rápida, simples e elegante.

## INTRODUCTION

The feeding through a gastrostomy tube is used to maintain or improve the nutritional status of patients with severe motor impairment of swallowing or obstruction due to cancer of the oropharynx and esophagus.

The first gastrostomy was done in the 19^th^ century, and Stamm technique, described in 1894, was considered standard for a long time to conduct prolonged enteral access. When the percutaneous endoscopic gastrostomy was described in 1980^3^, it has become a method of choice because of its speed and simplicity^5^^,^ 11 ^,^ 14. However, some clinical situations do not allow endoscopic access, either oropharyngeal and esophageal obstruction or anatomical abnormalities of abdominal cavity and stomach. Given these clinical scenarios surgical gastrostomy is the most used option. Morbidity related to gastrostomy range from 4% to 74% and mortality from 2.5% to 22%^1^^,^ 6 ^,^ 7 ^,^ 8 ^,^ 10 ^,^ 12 ^,^ 13 ^,^ 15. The complications are related to the incision and suture of the stomach with the laparotomy incision. 

In order to minimize the impact of these two factors, the author proposes a procedure that combines the direct visualization of the stomach obtained by laparotomy with the simplicity and less trauma offered by endoscopic gastrostomy.

## METHOD

### Surgical technique

**FIGURE 1:**
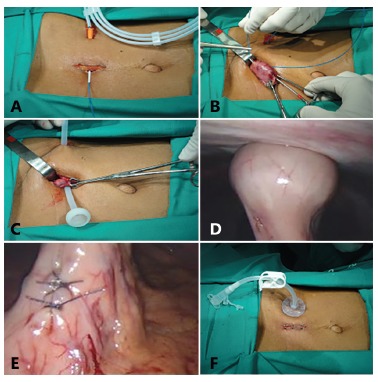
- Surgical technique of gastrostomy: A) guidewire passage perpendicularly to the abdominal wall under direct vision with its externalization through the incision; B) identification and seizing the gastric antrum with atraumatic forceps, gastrostomy location, spot-drilling perfuration in the distal antrum 5 cm from the site already undertaken to guidewire to be used for future passage of gastrostomy probe; C) seizure of the guidewire, connection with gastrostomy tube, smooth traction until the flange shaped element of the probe get into the stomach in close contact with the mucosa; D) after placement of the external fixing elements, the stomach probe is retracted against the abdominal wall - laparoscopic vision; E) suture of the gastric incision with nonabsorbable stitches far away from gastrostomy tube exit - laparoscopic vision; F) skin suture and placement of external elements finish the procedure

Patient is placed in the supine position under spinal anesthesia and antibioticprophylaxis. After antisepsis, initial incision of 2 cm is carefully carried out at midpoint between xiphoid process and umbilicus, keeping the round ligament to the right while the peritoneal opening is being done. Puncture location is chosen on the left hypochondrium and an incision of 3 mm is made, guided by gastric mobility. Through it will pass percutaneous puncture thick caliber needle and introduced a guidewire perpendicularly to the abdominal wall, under direct vision, externalized through the incision (Figure 1A). Next, is identified and seized the gastric antrum with atraumatic forceps; gastrostomy location is chosen; spot-drilling perfuration in the distal antrum 5 cm from the site already undertaken to guidewire to be used for future passage of gastrostomy probe, is done (Figure 1B). Follows the seizure of the guidewire; connection with gastrostomy tube; smooth traction until the flange shaped element of the probe internally put the stomach in close contact with the parietal peritoneum with part of the probe being exteriorized (Figure 1C). After placement of the external fixing elements, the stomach probe is retracted against the abdominal wall (Figure 1D). Suture of the gastric incision is made with nonabsorbable stitches far away from gastrostomy tube exit (Figure 1E). Skin suture and placement of external elements finish the procedure (Figure 1F).

Surgical gastrostomy using endoscopic gastrostomy devices was performed in 28 patients with oropharyngeal and esophageal cancer, with dietary obstruction.

## RESULTS

Of the 28 patients, six had minor complications (pain and/or abdominal distension, hyperemia and secretion by the gastrostomy tube). There were no major complications, such as off the gastrostomy tube, diffuse peritonitis, hemorrhage, wound dehiscence, need for reintervention or death. Patients started diet with reduced volume on the first day after surgery, passing to the standard diet from the second.

## DISCUSSION

The two main techniques of surgical gastrostomy, Stamm double string purse suture and Witzel's creating a serous tube, seek to involve the probe in gastric tissue, trying to avoid the leakage complications of gastric contents. The endoscopic gastrostomy with punctiform incision of the stomach and approaching it to the abdominal wall without suturing, produces lower morbidity and mortality or at least equal to the surgical gastrostomy. Is technically simpler, less traumatic and low cost^1^^,^ 2 ^,^ 6 ^,^ 7 ^,^ 8 ^,^ 9 ^,^ 10 ^,^ 12 ^,^ 13 ^,^ 15.

Gauderer on 2008^4^ described a technique called "hybrid" to use laparotomy access along with endoscopic procedure, describing the advantages of direct visualization of the stomach associated with simplicity and less traumatic surgical endoscopic technique. It was conducted in a group of children and adolescents with previous abdominal surgery and adhesions involving the stomach, making it difficult and unsafe making the endoscopic approach. The technique described here, because it was applied in adults with obstruction proximal to the stomach, used only open surgery without performing suture around the probe, similar to hybrid technology, relying on the approach and lock system already tested in the endoscopic procedure.

The advantages of this technique include: minimum laparotomy; easy to pull out the stomach cavity; punctiform incision in the stomach for passage of the probe guidewire and externalization, obviating the purse string suture in the stomach; probe with circular inner flange, used in endoscopic technique, allowing better traction and fixing the gastric wall to the parietal peritoneum.

The results in this group of patients showed low frequency of local complications, absence of major complications, especially when compared to the frequency of surgical gastrostomy complications reported in the literature, ranging from 4-74% for morbidity and 2.5-22% for mortality^1^^,^ 2 ^,^ 6 ^,^ 7 ^,^ 8 ^,^ 9 ^,^ 10 ^,^ 12 ^,^ 13 ^,^ 15. This procedure was performed by laparoscopy in two patients, easily and with good results. Laparoscopic view nicely illustrates the gastric pull-up to the abdominal wall (Figure 1D) and gastric suturing to insert the probe in distant location for gastrostomy tube externalization (Figure 1E).

The author has surgical activity in philanthropic hospital in SUS system in Brazil (free of medical/hospitalization charges) in city located in northeastern state of Bahia (poor region); therefore, he could not fail to discuss briefly the financing of material used in this proposal. Most patients with gastrostomy indication are SUS users, whose payment rules does not include the device for endoscopic gastrostomy.

Facing many complications related to the traditional surgical procedures, higher cost on diagnosis and in treatment complications, increased hospital stay and negative impact on patients outcome, the hospital administration permitted the use of the endoscopic material in this specific group of patients, to enable the method described herein.

Further studies comparing different surgical gastrostomy techniques with the current proposal may prove its equivalence.

## CONCLUSION

The surgical gastrostomy with minimal incision in the stomach to pull off the catheter using endoscopic gastrostomy devices, proved to be safe, easy to perform, less traumatic, quick, simple and elegant.
